# Supplementary material for: Hybrid Models and Biological Model Reduction with PyDSTool
Source: PLoS Comput Biol. 2012 Aug 9;8(8):e1002628. doi: 10.1371/journal.pcbi.1002628 (PMC3415397; doi:10.1371/journal.pcbi.1002628)
Supplement: Text S4 — Complete source code for the PyDSTool package (version 0.88.120504). Includes API documentation and help files linking to web pages. This file is identical to the current public release on Sourceforge.net. (ZIP) [file pcbi.1002628.s004.zip › PyDSTool/html/PyDSTool.Toolbox.optimizers.line_search.cubic_interpolation-pysrc.html]

xml version="1.0" encoding="ascii"?


PyDSTool.Toolbox.optimizers.line\_search.cubic\_interpolation


| Home | Trees | Indices | Help | PyDSTool |
| --- | --- | --- | --- | --- |

Package PyDSTool ::
Package Toolbox ::
Package optimizers ::
Package line\_search ::
Module cubic\_interpolation

[hide private]

[frames] | no frames]

# Source Code for Module PyDSTool.Toolbox.optimizers.line\_search.cubic\_interpolation

```
  1   
  2  # Matthieu Brucher 
  3  # Last Change : 2007-08-26 19:45 
  4   
  5  """ 
  6  Line Search with the cubic interpolation method with the computation of the gradient of the function 
  7  """ 
  8   
  9  import numpy 
 10  from numpy.linalg import norm 
 11   


12 -class CubicInterpolationSearch(object):


13    """ 
 14    Line Search with the cubic interpolation when the gradient of the function is provided 
 15    """ 


16 -  def __init__(self, min_alpha_step, alpha_step = 1., grad_tolerance = 1.e-6, **kwargs):


17      """ 
 18      Needs to have : 
 19        - a minimum step size (min_alpha_step) 
 20      Can have : 
 21        - a step modifier, a factor to modulate the step (alpha_step = 1.) 
 22        - the tolerance on the gradient (grad_tolerance = 1e-6) 
 23      """ 
 24      self.minStepSize = min_alpha_step 
 25      self.stepSize = alpha_step 
 26      self.gradtol =  grad_tolerance

 27   


28 -  def __call__(self, origin, function, state, **kwargs):


29      """ 
 30      Returns a good candidate 
 31      Parameters : 
 32        - origin is the origin of the search 
 33        - function is the function to minimize 
 34        - state is the state of the optimizer 
 35      """ 
 36      direction = state['direction'] 
 37      ak = 0. 
 38      if 'initial_alpha_step' in state: 
 39        h0 = state['initial_alpha_step'] 
 40      else: 
 41        h0 = self.stepSize 
 42   
 43      istop = -1 
 44   
 45      x0 = 0 
 46   
 47      f = lambda x : function(origin + x * direction) 
 48      df = lambda x : numpy.dot(function.gradient(origin + x * direction), direction) 
 49   
 50      def f_and_df(x, fv = None): 
 51          if fv is None: 
 52            return f(x), df(x) 
 53          else: 
 54            return fv, df(x)

 55   
 56      f0 = None#TODO: extract f0 from state 
 57      f0, df0 = f_and_df(x0, f0) 
 58   
 59      #TODO: rename something, step is inconvenient 
 60      xtol = self.minStepSize 
 61   
 62      #if norm(df0) < gradtol: return origin + x0 * direction 
 63      h = h0 
 64   
 65      m1 = x0 
 66      f1 = f0 
 67      df1 = df0 
 68   
 69      while True: 
 70          if df1 > 0: h = -abs(h) 
 71          else: h = abs(h) 
 72   
 73          while True: 
 74              #iter += 1 
 75              #if iter > maxiter: istop = 0; break 
 76              m2 = m1 + h 
 77              f2, df2 = f_and_df(m2) 
 78              if df1 * df2 <= 0: 
 79                break# in the origin algorithm here is '<' but I think here should be '<=' 
 80              h *= 2.0 
 81              m1, f1, df1 = m2, f2, df2 
 82   
 83          if h>0: 
 84            a, b = m1, m2 
 85          else:  
 86              a, b = m2, m1 
 87              f1, f2 = f2, f1 
 88              df1, df2 = df2, df1 # in the origin algorithm ithe line is absent but I think it should be present elseware error occures 
 89   
 90          S = 3.0 * (f2-f1) / (b-a) 
 91          z = S - df1 -df2 
 92          w = numpy.sqrt(z**2 - df1*df2) 
 93          z = 1.0 - (df2 + w + z) / (df2 - df1 + 2*w) 
 94          m1 += (b-a)*z 
 95   
 96          f1, df1 = f_and_df(m1) 
 97          h /= 10.0 
 98   
 99          if abs(m1-m2) <= xtol: 
100            istop = 3 
101          elif norm(df1) <= self.gradtol: 
102            istop = 2 
103          if istop>0: 
104            break 
105   
106      state['alpha_step'] = m1 - x0 
107      return origin + m1 * direction

108
```

  


| Home | Trees | Indices | Help | PyDSTool |
| --- | --- | --- | --- | --- |

|  |  |
| --- | --- |
| Generated by Epydoc 3.0.1 on Fri May 4 15:24:18 2012 | http://epydoc.sourceforge.net |
